# Light-field-driven electronics electronics in the mid-infrared regime: Schottky rectification

**DOI:** 10.1126/sciadv.abj5014

**Published:** 2022-06-03

**Authors:** Maria T. Schlecht, Matthias Knorr, Christoph P. Schmid, Stefan Malzer, Rupert Huber, Heiko B. Weber

**Affiliations:** 1 Chair for Applied Physics, Friedrich-Alexander-Universität Erlangen-Nürnberg (FAU), D-91058 Erlangen, Germany.; 2 Department of Physics, University of Regensburg, D-93040 Regensburg, Germany.

## Abstract

The speed of an active electronic semiconductor device is limited by *RC* timescale, i.e., the time required for its charging and discharging. To circumvent this ubiquitous limitation of conventional electronics, we investigate diodes under intense mid-infrared light-field pulses. We choose epitaxial graphene on silicon carbide as a metal/semiconductor pair, acting as an ultrarobust and almost-transparent Schottky diode. The usually dominant forward direction is suppressed, but a characteristic signal occurs in reverse bias. For its theoretical description, we consider tunneling through the light-field–modulated Schottky barrier, complemented by a dynamical accumulation correction. On the basis only of the DC parametrization of the diode, the model provides a consistent and accurate description of the experimentally observed infrared phenomena. This allows the conclusion that cycle-by-cycle dynamics determines rectification. As the chosen materials have proven capabilities for transistors, circuits, and even a full logic, we see a way to establish light-field-driven electronics with rapidly increasing functionality.

## Teaser

Driving a Schottky diode with mid-infrared light fields results in rectification far beyond usual electronic frequencies.

## INTRODUCTION

**Fig. 1.**
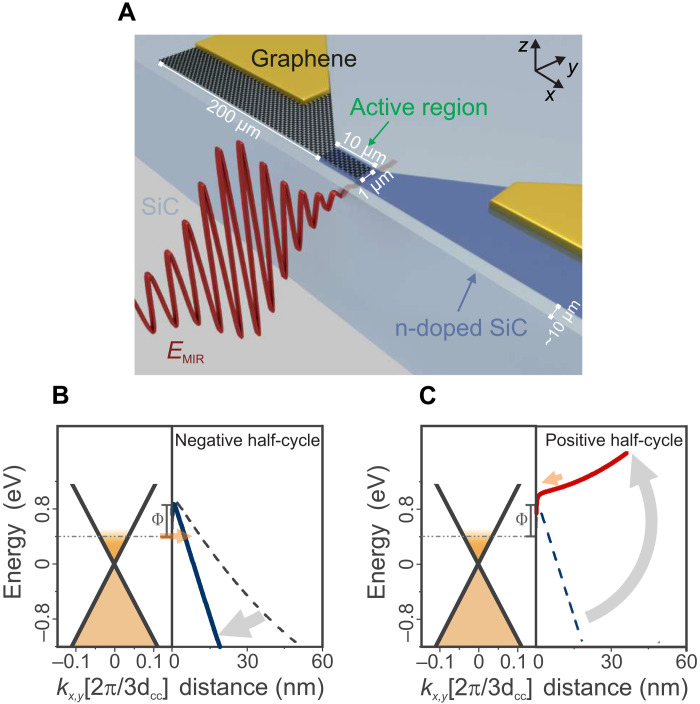
Schematic of the experiment. (**A**) Graphene as a metal and SiC as semiconductor form a Schottky diode in the *z* direction. A MIR pulse is applied from the sidewall such that its electric fields are parallel to the intrinsic electric field of the diode. (**B** and **C**) Energy scheme of the graphene/SiC interface. The electric field of the MIR pulse tilts the potential landscape in the space charge region accordingly, alternating between an extremely thin barrier (B) and field inversion (C). Electrons are injected from a graphene’s Fermi level via tunneling through the Schottky barrier.

Light-field electronics aims at controlling currents with electromagnetic wave cycles on time scales much faster than conventional electronics (*1*–*12*). For increasing functionality, miniaturization, and complexity (*13*–*16*), implementation of this concept into electronic devices and circuits is the next and necessary step. Among the desiderata for studying light-field electronics in real devices, robustness and transparency are the most important. The absence of plasmons is also helpful for clarifying the intrinsic mechanisms, although they might enhance light-field sensitivity considerably in real devices (*16*, *17*). We propose Schottky diodes based on epitaxial graphene for this purpose. This material, grown on 4H silicon carbide (SiC), is essentially an atomically thin metal and has proven to be ultrarobust (*4*). When the substrate is not chosen as an insulator but rather as a semiconductor, graphene on SiC is a monolithic metal-semiconductor system (see Fig. 1A), in which diodes, transistors, and logic circuits can be implemented (*18*, *19*). More specifically, the DC characteristics of n-type 4H SiC and the as-grown graphene (*20*) behave like a textbook Schottky diode with a barrier height of Φ = 0.35 eV (see Fig. 1B; DC parameters are given in table S1). This behavior remains unchanged when the characteristics are recorded in the high-gigahertz/low-terahertz regime (in antenna-coupled devices), even the current-voltage *IV* characteristics can be reproduced with high fidelity (*21*). However, in this frequency range, the *RC* limitation becomes apparent (series cutoff frequency ν_cutoff_ ≈ 100 GHz). When choosing such devices for light-field electronics, these extrinsic constraints on driving the diode can be overcome, and the question of whether there are intrinsic limitations to rectification arises.

## RESULTS

We expose the epitaxial graphene Schottky diode to electromagnetic radiation with its fast-oscillating electric field and measure its time-integrated current response. Here, we address driving frequencies in the far–to–mid-infrared (MIR) spectral region, where single-photon and multiphoton excitations remain well below the Schottky barrier and thus contribute little to the current through the diode (the unique band structure of graphene allows such processes only for photon energies above ~1.6 eV). We focus first on data recorded at a driving frequency of 42 THz. The picture is complemented by data at 18 and 78 THz. In this spectral range, SiC has a Reststrahlen band (*22*–*24*), the influence of which is discussed in the Supplementary Materials (*25*). For light-field operation, an out-of-plane electric field is required. This is conceptually different from previous experiments, where the optical fields were polarized in the graphene plane (*4*, *5*, *26*). To ensure this, we opted for cutting the chip next to the Schottky junction, such that phase-locked MIR pulses can be applied to the Schottky junction region from the sidewall (see Fig. 1A). When the polarization of the incident pulse is chosen perpendicular to the graphene sheet, its oscillatory electric field adds to the static electric field associated with the space charge region (*27*). Alternatively, the light field can also be chosen parallel to the graphene sheet. As we will see, this is helpful for discriminating field-driven effects from thermal ones.

Conceptually, all-optical methods could resolve ultrafast time scales (*17*, *28*). However, in a Schottky diode, the volume threaded by light-induced currents is extremely small (a few nanometers in thickness and a few square micrometers in area), and hence, their optical signal is barely detectable. Any electrical detection method, however, integrates over the time evolution of an electromagnetic pulse. One class of experiments uses the pulse’s carrier-envelope phase as an indicator of field-driven effects (*4*, *28*–*30*), but the strength of these effects critically depends on a low number of electric field cycles in the temporal envelope of the driving pulse. We propose here an electrical method with an accurate measurement of the charge per pulse, where the static bias voltage at the device is used as a well-controlled parameter, similar to previous measurement concepts in ultrafast scanning tunneling microscopy (*11*). It will turn out that this information is sufficient to identify rectification and even learn about subcycle dynamical processes. All experiments are carried out under ambient conditions.

**Fig. 2.**
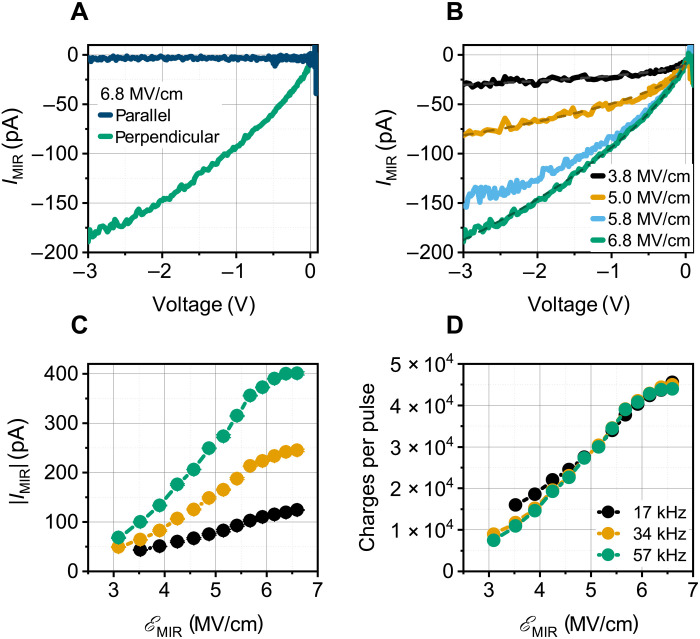
Light-field-induced currents at a driving frequency of 42 THz. (**A**) The comparison between pulses polarized parallel and perpendicular to the graphene plane reveals a field-driven effect. (**B**) For each pulse amplitude, sublinear shape is observed, which demands for a transient charge correction (Eq. 3). (**C**) The MIR-induced current rises quadratically with the maximum electric field up to 6 MV/cm^2^ at a bias voltage of −1 V, where a subtle saturation is observed. (**D**) A variation of the repetition rate has little influence on the signal per pulse, from which we infer that the effect is related to a single-pulse phenomenon.

Figure 2 displays results of the electrical signal upon application of MIR pulses at ν_center_ = 42 THz. This is a spectral regime in which ~20% of the incident light is reflected, and absorption of the incident radiation can be expected within 50 μm (Reststrahlen band; cf. fig. S2C). The Schottky diode, being at only 10 μm in depth, is therefore penetrated by the MIR radiation. To determine the fraction of the electric field E_MIR_(*t*) that acts in the device region, finite-difference time-domain simulations with Lumerical were carried out for the given geometry, and screening within graphene was neglected. In Fig. 2 (A and B), the abscissa is the static voltage applied to the Schottky junction in reverse bias direction (the associated DC electron current flows from graphene to SiC). The ordinate represents the MIR-induced current response *I*_MIR_ in addition to *I*_DC_. In Fig. 2A, the data compare *I*_MIR_ for a polarization parallel to the graphene plane with that perpendicular to the graphene plane. The parallel case, appearing as a straight line, can be traced back to the temperature dependence of the saturation current and will be discussed below. When rotating the polarization by 90° so that the electric field of the MIR pulse adds to the static field of the Schottky diode, a substantial current contribution arises, and the characteristics become sublinear. Its polarization dependency is an evident indicator for a field-driven effect.

Figure 2B shows this signal for four different maximum electric fields ranging from 3.8 to 6.8 MV/cm under otherwise constant conditions. All curves exhibit a sublinear dependency, which is neither compatible with the simple description of tunneling currents nor with thermionic currents (see the Supplementary Materials). One may suspect that the effect is based on multiphoton excitations in graphene that constitute the current. With the given photon energy (0.17 eV), the given Schottky barrier height (ϕ = 0.35 eV), and the band structure of graphene (see Fig. 1B) (Fermi level of 0.45 eV above the Dirac cone), at least a 10-photon excitation must be assumed, for which a high power law (similar to *E*^20^) would be expected. Figure 2C displays a closer look on the measured MIR electric field dependence for a constant bias voltage *V* = − 1 V at three different repetition rates. It shows that *I*_MIR_ rises proportionally to *E*^2.5^ up to about 6 MV/cm. Such effective exponents are often observed in crossover regimes from perturbative to nonperturbative light-matter interaction (*8*). For higher field strength, saturation is observed, which is in obvious contradiction to multiphoton-dominated effects.

The current rises linearly with the number of pulses (per chopping interval). The latter suggests that there is an equal number of charge carriers being induced by each pulse (see Fig. 2D). The Keldysh parameter at a field strength of 6 MV/cm is approximately 0.8, indicating a strong field regime dominated by field-induced tunneling processes.

## DISCUSSION

For the interpretation of these data, we seek for the most instructive model that provides a consistent description of the phenomena. We start off with the textbook description (*31*, *32*) of a tunneling current density *j* through a Schottky barrier in the static case

$$\begin{array}{cc} j= & \frac{A^{*}T^{2}}{k_{B}T}(\int_{E_{\text{Fs}}}^{q\Phi_{\text{bn}}}F_{M}T(E)(1-F_{S})\;\mathrm{dE}-\\ & \int_{E_{\text{Fs}}}^{q\Phi_{\text{bn}}}F_{S}T(E)(1-F_{M})\;\mathrm{dE}) \end{array}$$ (1)

with the Fermi distribution function *F*_M, S_ for the metallic and semiconducting side, respectively. *T* denotes the temperature, *E* denotes the energy, and A* represents the reduced effective Richardson constant, and

$$T(E)=\text{exp}(-2\int_{0}^{d_{\mathrm{dz}}}\sqrt{\frac{2m_{e}(\Phi_{{ℰ}_{0,V}}(x)-E)}{\hbar^{2}}}\mathrm{ds})$$ (2)

is the transmission function (in Wentzel-Kramers-Brillouin approximation).

**Fig. 3.**
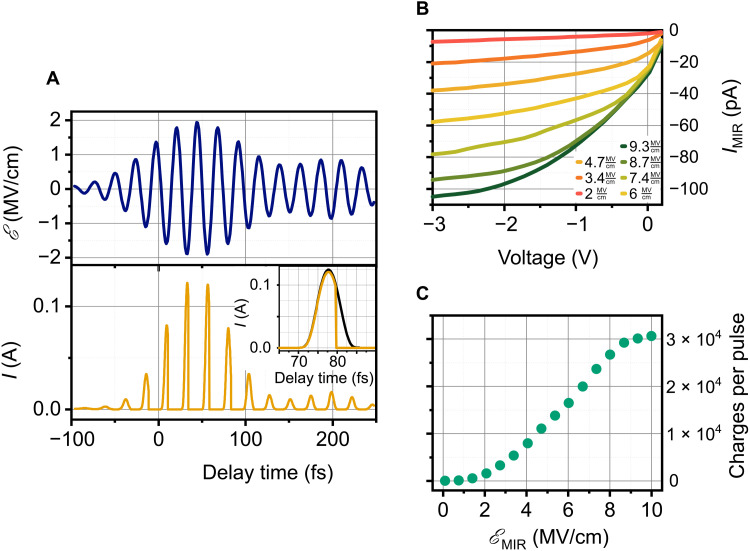
Calculated response to the 42-THz MIR pulse. (**A**) Temporal evolution of the electric field of the MIR pulse centered at 42 THz (top) and the calculated tunneling current according to Eq. 1 with transient charge correction. The inset depicts the influence of the latter; orange line indicates with consideration of the return current, and the black line indicates without consideration of the return current. The triangular area thus marks tunneled charges that do not contribute to the overall current because they will be thrown back within the next half-cycle. (**B**) Calculated bias and amplitude dependence of the rectified current. (**C**) Calculated charge per pulse. All parameters were extracted from DC diode parameters (cf. table S1) without any fit parameters.

The tunneling potential Φ_ℰ_0, *V*__ depicts the barrier shape under the influence of an external electric field E_0, *V*_ including image force corrections (Schottky effect). We further assume that the MIR electric field adds onto the static bias electric field such that the tunnel barrier is modulated by E_0, *V*_ + E_MIR_(*t*); see, for example, Fig. 3A for the 42-THz pulse. Note that the MIR fields are so strong that the barrier is switched on and off in the course of each light cycle (cf. Fig. 1B). The current as a function of time, *I*(*t*), can be readily calculated, as displayed in Fig. 3A. Note that the tunneling current is completely suppressed during the positive electric field cycle of the MIR pulse. Hence, the nonlinearity of the tunnel process rectifies and provides intense current spikes when E_0, *V*_ + E_MIR_(*t*) reaches the diode’s breakdown electric field. If we stopped the description here, then we would obtain an exponential increase in the MIR-induced tunneling current with increasing bias voltage, in contrast to the oppositely curved experimental characteristics (Fig. 2, A and B).

This qualitative discrepancy evidences that the light field–driven Schottky device uses critical dynamics beyond a quasi-static model. To capture the essence of these new dynamics, we extend Schottky’s model beyond the original time-independent description. Within the short time scales of light cycles, the charge that has just passed the thin barrier is not absorbed as in DC operation but remains present in the space charge region and acts back on the electrostatics. We express this by summing up charges via a time-dependent tunneling current density *j*_reverse_(*t*) in the course of a pulse (tunneling time is assumed to be instantaneous). Once tunneled, this charge experiences the MIR-modulated electrostatics again, leading either to acceleration into the SiC half space or to a return into graphene. The latter is expressed by *j*_return_(*t*) and has its maximal contribution for those charges that tunnel toward the end of a negative cycle of E_MIR_(*t*) and are immediately thrown back into the graphene sheet by the subsequent positive cycle. We model this dynamic evolution via semiclassical equations of motion (*33*) (for a detailed description, see the Supplementary Materials). The result is a “dynamical Schottky effect (DSE)” electric field contribution evolving during the pulse

$${ℰ}_{\text{DSE}}(t)=-\frac{1}{\epsilon_{0}\epsilon_{r}}[\int_{0}^{t}j_{\text{reverse}}(\tau )\;d\tau -\int_{0}^{t}j_{\text{return}}(\tau^{*})d\tau^{*}]$$ (3)

that adds to the electric field, leading to E_total_(*t*) = E_0_ + E_MIR_(*t*) + E_DSE_(*t*), and is consistently smaller than E_0_ + E_MIR_(*t*). In contrast to the original Schottky effect, which describes the image charge lowering of the barrier and is valid at any time (*31*), DSE is an electric field correction due to accumulated charges in the space charge region. Therefore, E_total_(*t*) depends critically on the pulse shape and on the electron dynamics of the electrons after tunneling.

As all quantities, including the pulse shape, are known, we can calculate the field-driven current as a function of time for a given pulse and applied (reverse) bias voltage (see Fig. 3A). The result is displayed in Fig. 3 (B and C): The shape of the experimental curve with their characteristic sublinear dependence is qualitatively very well reproduced; a factor as small as ~2 has to be applied to match the amplitude accurately (all parameters are determined from DC characteristics; see table S1). Whether the small deviation originates in nonidealities of the experiments or may indicate additional mechanisms will be a topic of future investigations. Figure 3C displays the calculated field amplitude dependence of the charges per pulse, in analogy to Fig. 2D (both evaluated at *V* = –1 V). Not only the quadratic shape is well reproduced by the calculation, but also the saturation at 6 MV/cm is reproduced without any further assumptions.

**Fig. 4.**
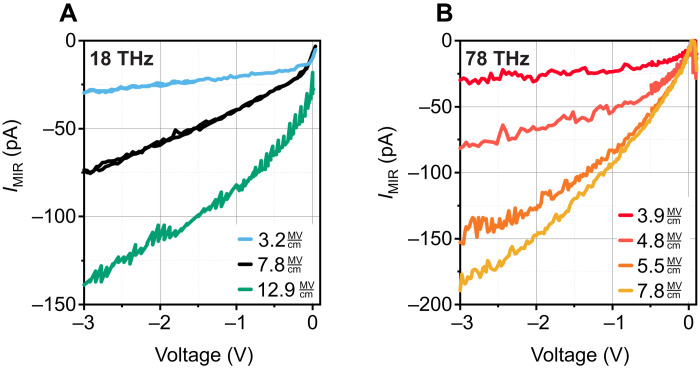
Light-field-induced currents at further carrier frequencies. (**A**) At 18 THz, at the center of the Reststrahlen band, the phenomena observed and described for 42 THz occur only at higher field amplitudes. (**B**) At 78 THz, above the Reststrahlen band, the phenomenology is again quite similar. The data can consistently be described with one model and one parameter set (obtained from DC parameters).

Figure 4 shows data obtained on the same sample at two further carrier frequencies in the MIR: At 18 THz, i.e., at the center of the Reststrahlen band, higher amplitudes are required to obtain the same shape of the light field–induced currents, as expected for the stronger damping (Fig. 4A). For frequencies well above the Reststrahlen band (78 THz), again, very similar phenomena are observed (Fig. 4B). A theoretical description based on the very same DC parameters of the sample (cf. table S1) gives again a fully consistent description, spanning from DC over the entire MIR range under investigation (see the Supplementary Materials). At even higher driving frequencies, with Keldysh parameters strongly exceeding unity, multiphoton excitations are expected to dominate over tunneling. Below this suspected limitation, we assess the Schottky theory with the proposed electric field correction E_DSE_(*t*) as a predictive theory.

We lastly discuss the behavior in forward bias. Here, the DC current is thermionic, but we find no polarization-dependent MIR response (see fig. S2A). The absence of a polarization-dependent and hence field-driven response is in full agreement with the earlier observation of an *RC* roll-off at ~100 GHz (*21*), and the remaining signal points to a slow thermal effect. We could fully explain the data by a heating effect on time scales that average over the pulse train (see the Supplementary Materials).

Together, we demonstrated the light-field operation of a Schottky diode, showing rectification of a few-cycle MIR pulse in the reverse bias regime. The data, obtained for several frequencies, can be consistently understood by the Schottky model, originally developed for the DC case, when an additional transient electric field correction is introduced. This “DSE” is specific to the fast time scales of light cycles. We can describe rectification and electrical characteristics from DC to the infrared spectral range with only one parameter set. The rectification operates in the reverse direction because it stems from the breakdown at high negative bias. While *I*_MIR_ appears small when time-averaged, its peak levels exceed the background DC tunneling current by orders of magnitude.

The results not only show consistent experiments and the theory of Schottky rectification in the MIR. They demonstrate a material system that has all the prerequisites for a platform for light field–driven electronics: epitaxial graphene on silicon carbide. Having demonstrated the MIR rectification of the almost-transparent Schottky diode, the way is paved to add further functionality, either by a plasmonic environment or waveguides toward ultrafast circuitry.

## MATERIALS AND METHODS

### Sample fabrication

Samples were fabricated on a 6 mm–by–6 mm 4H high-purity, semi-insulating SiC chip from Cree Inc. The semi-insulating material was partially implanted with nitrogen in a two-step process to define the Schottky and the ohmic contact regions. Below the Schottky contact, the implantation concentration was low at 1 **×** 10^17^cm^−3^. Beneath the Schottky contact, a current spreading layer was included to decrease the sheet resistance of the sample. There, the implantation concentration rose to 1 **×** 10^18^cm^−3^. In a second implantation step, the ohmic contact region was implanted with a dopant concentration of 1 **×** 10^19^cm^−3^. The implantation was performed at room temperature. Subsequently, the sample was annealed, and graphene was grown on the Si-terminated surface (*20*). Last, the contact regions were defined by standard lithography methods. The unwanted graphene areas were removed in an oxygen plasma. After the electrical characterization, the SiC chip is sliced into 1 mm–by–1 mm chips each containing one Schottky diode. This way, the distance of the SiC edge and the Schottky contact amounts to only 10 to 20 μm. An artist’s view of the device is presented in Fig. 1A.

### Experimental setup

The generation of the MIR driving fields with center frequencies at 42 and 78 THz closely follows the approach described in (*34*). The laser fundamental of an Yb:KGW amplifier (center wavelength, 1026 nm; pulse energy, 14.3 μJ) and the output of an optical parametric amplifier drive a difference frequency mixing process in LiGaS_2_. For measurements with perpendicular polarization, type II phase matching in the *XY* plane was chosen, while for parallel polarization, we use a crystal cut for type I phase matching in the *XZ* plane. For both polarizations, we obtain pulse energies of up to 0.3 μJ at 42 THz and up to 0.6 μJ at 78 THz. The time-averaged MIR power can be adjusted by varying the repetition rate of the laser output up to a maximum of 170 kHz. A pair of holographic wire grid polarizers allows to adjust the peak electric field through rotation of the first polarizer, while the second polarizer ensures that the polarization at the sample is maintained. Electro-optic sampling is performed in a 5-μm-thick GaSe crystal contacted onto a diamond substrate. Pulses centered at 18 THz were derived from a dual-stage optical parametric amplifier driven by a Ti:Sapphire laser at a 3-kHz repetition rate. Measurements were performed at ambient atmosphere. The electrical measurement was performed by applying a DC voltage to the sample in addition to the light field of the MIR pulse. A 10-kilohm resistor in series was used for measuring the rectified current response induced by the MIR pulses, using a lock-in detection scheme (800-Hz modulation frequency). The *RC* roll-off of the diode with graphene leads is estimated to be ~0.3 GHz.
