# Supplementary material for: Light-field-driven electronics electronics in the mid-infrared regime: Schottky rectification
Source: Sci Adv. 2022 Jun 3;8(22):eabj5014. doi: 10.1126/sciadv.abj5014 (PMC9166296; doi:10.1126/sciadv.abj5014)
Supplement: Supplementary file 1 — Supplementary Text Figs. S1 to S6 Table S1 [file sciadv.abj5014_sm.pdf]

Science Advances
AAAS

Supplementary Materials for

Light-field-driven electronics in the mid-infrared regime: Schottky rectification

Maria T. Schlecht *et al.*

Corresponding author: Heiko B. Weber, heiko.weber@fau.de

Sci. Adv. **8**, eabj5014 (2022)
DOI: 10.1126/sciadv.abj5014

This PDF file includes:

Supplementary Text
Figs. S1 to S6
Tables S1

Supplementary Text

Parameters extracted from DC characteristics

Fig. S1 presents an IV curve of one of the devices that was used to measure the field-induced current in reverse direction. The barrier shape of a Schottky diode as a function of distance *x* is given by

$$\Phi_0(x) = \Phi_b - \frac{q^2 N_D}{\epsilon_r \epsilon_0}\left(d_{dz}x - \frac{x^2}{2}\right) - \Phi_{IF} \text{ (S1)}$$

Here, N_D is the doping concentration of the semiconductor, d_{dz} is the depletion zone and Φ_{IF} is the image force potential. The DC characteristics of such a device can be modelled by the Schottky model including thermionic emission and tunneling current. Here, the reduced, effective Richardson constant A* was determined from a temperature-dependent IV measurements of a structurally identical device. Afterwards, A* was employed to fit the IV characteristic of the Schottky diode that was used during the experiments described in this paper. The fit curve included thermionic emission current and tunneling current. The latter was calculated according to equation (1) and (2), while the thermionic emission current I_{th} was determined by the well-known diode equation:

$$I_{th} = A\,A^{*}T^2 \exp\left(-\frac{q\,\Phi}{k_B T}\right)\left(exp\left(\frac{qV}{k_B T}\right) - 1\right) \text{ (S2)}$$

Here, *A* denotes the area of the Schottky diode and *V* stands for the voltage that is applied to the ideal diode. Fit parameters were the ideality factor of the diode *n*, the doping concentration of the space charge region *N* and the barrier height ϕ. These diode parameters are presented in table S1.

Forward-bias signal

We consistently find a characteristic signal in forward direction, see Fig. S2, for all devices under study. We recall the fact that in this spectral regime partial absorption of the incident radiation can be expected (Reststrahlen band, cf. inset Fig. S2 c). The abscissa in Fig. S2 is the static voltage applied to the Schottky diode. The ordinate represents the MIR-induced current response I_{MIR} in addition to I_{DC}. Fig. S2a shows the polarization dependency of the MIR induced signal for a few-cycle pulse with a center frequency of 42 THz. While the signal shows a clear polarization dependency in reverse bias, no polarization dependency was observed in forward direction (the associated DC electron current flows from SiC to graphene). Here, first an increase, then a sign change and then a linear negative current response can be seen. When increasing the average MIR power, this reentrant behavior is amplified without

changing its shape (see Fig S2 b, $\nu_{\mathrm{center}} = 38$ THz). Interestingly, the voltage where the sign of I_{MIR} flips remains unchanged.

Obviously, there are two regimes in the current response in forward direction presented in Fig. S2, the initial increase and the (counteracting) linear decrease. This behavior has been observed at 5 different samples, at 7 different MIR frequencies and raw data are available in a data repository (DOI 10.22000/414). Further, we have studied in detail the dependence of I_{MIR} when the focus is displaced from the center of the Schottky diode. The current response of the diode decreases only weakly with increasing distance of the focus, an observation that suggests a nonlocal physical mechanism.

Thermal simulations

We performed extensive simulations using the multiphysics simulations tool COMSOL. The geometry is simplified as a SiC cuboid (SiC has an excellent heat conduction of $\lambda = 3{,}7 \frac{\mathrm{W}}{\mathrm{cm\,K}}$). The absorption of the MIR pulse train is modeled by a static heat source of 20mW in a quarter-ellipsoid volume that corresponds to the absorption volume (width determined by the experimentally determined focus: 50µm; penetration depth at 29.4 THz: 20µm). A metallic contact at 1 mm distance serves as a heat sink. The outcome of the simulation is a temperature profile that allows determining the (static) temperature rise due to the MIR illumination at various points of interest (see Fig. S3 a). An analysis of the temperature rise/fall times justifies ignoring timescales smaller than the chopping rate (see Fig S3 b), in particular the pulse train is not temporarily resolved ($\tau_{\mathrm{repetition}} = 5.9$ µs). Even under the given illumination of 20mW, the temperature rise is only 0.4 K, if the pulse is completely absorbed within the first 20µm, a fact that results from the excellent heat conduction of monocrystalline 4H SiC. On the first sight, this small temperature rise is not expected to have significant influence of the rather T-insensitive Schottky diode device. However, a closer look reveals that here is a weak temperature dependence of the Schottky diode. The diode conducts more current at higher temperature, which effectively provide a positive heat coefficient to the overall resistance. Moreover, the serial resistances are T sensitive: about 95% of the serial resistance is contributed by graphene leads with their positive heat coefficient (20). The remaining 5% are contributed by electrical pathways through SiC, i.e. with negative heat coefficients, which provide only a small correction. Comparing the simulation with the MIR-induced currents (cf. Fig. S2), the first contribution appears as an initial increase of the MIR-induced currents up to a plateau (see Fig. S3c), the sum of the two contributions to the serial resistance occur for higher positive voltages as a straight line towards negative I_{MIR}, see Fig S3c. Note, that the signal shape can be accurately assigned to a temperature rise. Fitting the data presented in Fig S2 with this model, reveals that the temperature of the device rises by 20mK due to the MIR pulse. This small temperature increase leads to a significant current response of the device in forward direction. Hence, the MIR induced phenomena in forward direction are consistently described by a thermal model. Yet, the temperature increase detected in the course of the experiment (20 mK) differs from the expected on (0.4 K). This hints, that the phonon-coupling is reduced at the crystal facet used in this experiment.

Thermal signal in reverse direction

The thermal model describes the current response of the Schottky diodes in forward direction. In reverse direction, it predicts a linear increase of the current as soon as the voltage exceeds -0.1 V (see Fig S4a for a temperature increase of 10mK). This linear increase is caused by the barrier lowering of the diode due to the Schottky effect. The serial resistance does not contribute to the current response in reverse direction. Therefore, the sublinear (approx. square-root like) behavior that was observed in the experiments in reverse direction cannot be explained by the temperature increase due to phonon-photon coupling. Furthermore, pure tunneling current would rise superlinearly as a function of the applied bias voltage (see Fi S4b).

Model for the semi-classical propagation after tunneling

After tunneling the electrons are accelerated by the electric field at the interface $\mathcal{E}_{total}(t)$. Their acceleration is treated semi-classically:

$$k_z(t) = \frac{q}{\hbar}\int_{t^*}^{t} E_{\text{total}}(t)dt \ + k_{0z} = \frac{p_z(t)}{\hbar} \text{ (S3)}$$

It is assumed that the initial wave vector k_{0z} of the electrons equals zero. Furthermore, electron-electron interactions are neglected and the electron package is treated like a delta distribution of electrons after the tunneling process. The position of the electrons as a function of time is then given by:

$$z(t) = \frac{1}{m_e}\int_{t_0}^{t^*} p_z(t)dt^* \ + z_0(t_0) \quad \text{(S4)}$$

Here, $z_0(t^*)$ is the position of the electrons after the tunnelling process. Due to the oscillating electric field of the MIR pulse, electrons are accelerated back towards graphene. The static field contributes to this close to the interface, due to the Schottky effect. These electrons interact with the electrons within the graphene and do not contribute to the total current. Yet, the electron temperature within the graphene rises.

Pulse shape comparison in the rectification regime: short and long pulses

According to the calculations, this effect appears under both strong electric field oscillations (such that $\mathcal{E}_{\text{total}}(t)$ reaches the Zener-like breakdown electric field) and high enough frequencies. For its illustration, we chose a single-cycle light pulse and compute its effect on the photocurrent characteristics in reverse bias. Here, charge is transmitted via the diode, but the charge build-up has little consequences. In contrast, when a continuous-wave (CW) MIR signal with the same amplitude is entered into the simulation, the charge builds up cycle by cycle, such that its decay becomes important. We chose a description $\approx exp\left(-\frac{t}{\tau}\right)$ for the decay with a characteristic timescale τ. For the CW case, a stationary state will be established, in which charge buildup according to (3) and decay are balanced. These considerations enclose the regime, in which the effect becomes apparent: the low frequency limit is given by τ^{-1}. We propose that it is related to the transit time per electron through the space charge region $\tau = \frac{d}{v_{\text{sat}}} \approx 250$ fs at 0V. We suspect that this is the correct description in the infrared up to light frequencies where multi-photon-absorption within graphene becomes important.

Rectification at 18 THz and 78 THz

Figure S5 shows the calculated curves of the current signal in reverse direction for pulses with center frequencies of 18 THZ and 78 THz.

Carrier envelope phase (CEP) dependency

Figure S6 shows the measured CEP dependency of the MIR induced current at a center frequency of 18 THz. The current shows a clear CEP dependency. However, the mean pulse power also shows slight delay-dependent variations, possibly due to nonlinearities in the difference frequency mixing crystal. Due to the proximity to the Reststrahlenband and the consequent heating of the crystal, it is not possible to fully disentangle the measured current as it consists of both a thermal contribution and a field dependent contribution. Based on the measurements performed at 37 THz we expect the following temperature dependency of the system:

$$\Delta T = \frac{-0.00181P+0.0059P^{2}+0.00753\ ^{3}}{P_{reference}} .$$

$P_{reference}$ amounts to 15 mW. Note that the absorption coefficients at 18 THz and at 37 THz are both approximately 0.6 and thus it is fair to assume that the dependency is also valid at 18 THz. If the mean MIR power rises from 5.2 mW to 5.5 mW a current increase of 2.5 pA ± 1.0 pA is expected. Furthermore, according to the model presented in this paper, the field dependent current is expected to rise by approximately 1 pA if the CEP is changed. Consequently, considering thermal and field induced current contributions, an amplitude change of approximately 3.5 pA is expected which agrees well with the measurement. Note that the model of the field induced current slightly underestimates the MIR induced current.

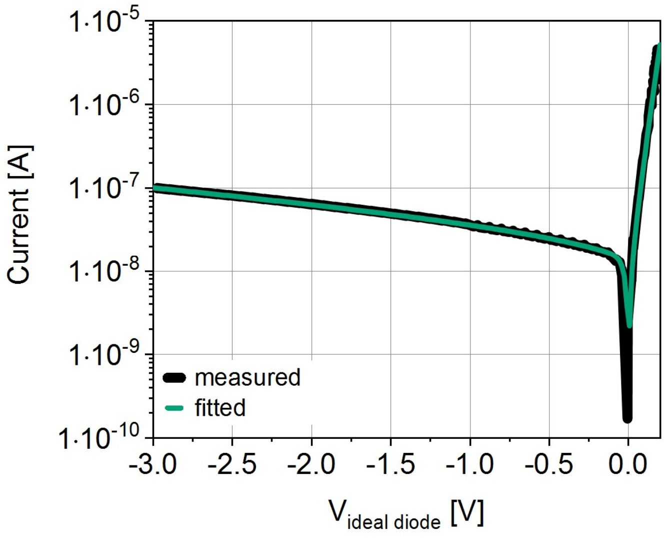

Fig. S1: Evaluation of the DC IV-characteristics: The DC IV characteristics of an ideal MLG/SiC Schottky diode can be perfectly modelled by considering thermionic emission current and tunneling current. A serial resistance was not taken into account.

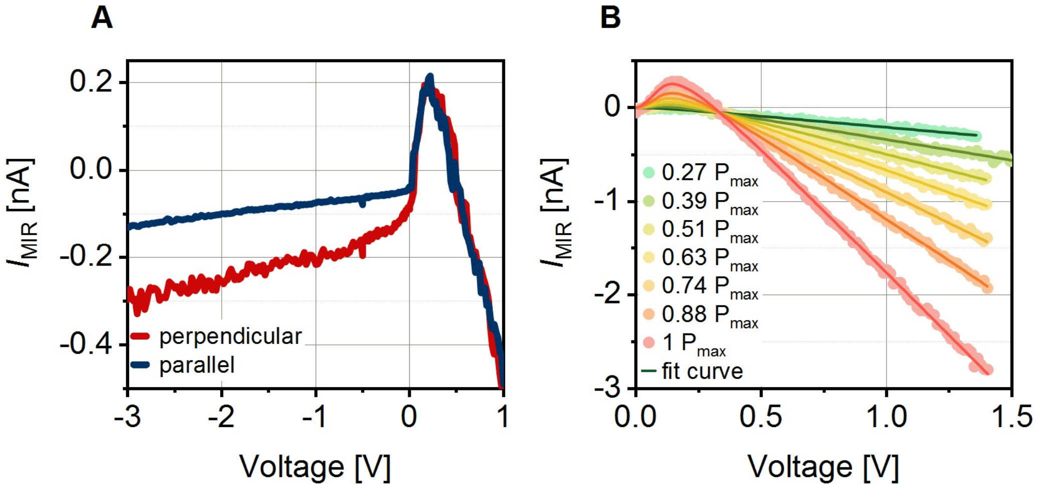

Fig. S2: Thermal signal in forward direction: (A) While the polarization dependency in reverse direction indicates a field-driven current signal, no polarization dependency can be observed in forward direction. (B) Thermally induced current as a function of the mean MIR power. The maximum mean power amounted to 20 mW. The lines resemble a fit of the thermal model presented in the following section.

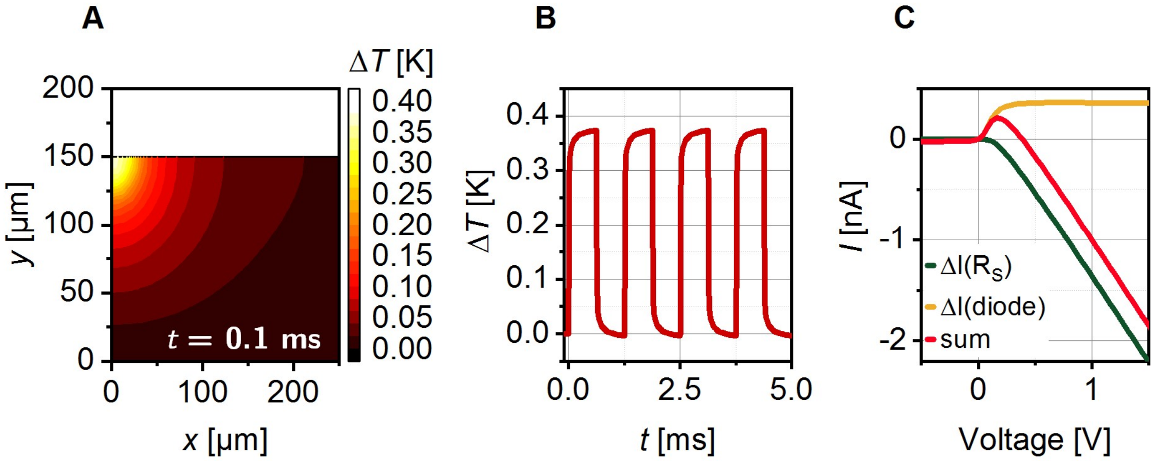

Fig. S3: Thermal simulations: (A) Spatial distribution of the temperature increase in the SiC chip when the sample is heated by the MIR pulse. The simulations were carried out with the FEM simulation tool COMSOL. (B) Temporal evolution of the temperature increase at the Schottky diode. Here, the MIR beam is periodically blocked with a frequency of 800 Hz (1.25 ms). (C) Calculated *IV* response of the ideal Schottky diode, the serial resistance and the whole device when the sample is heated by 10mK.

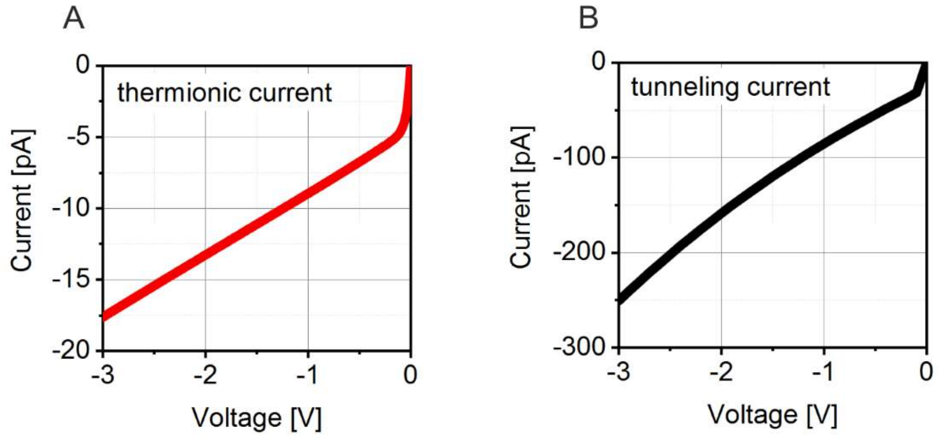

Fig. S4: Modelled MIR induced current in reverse direction: (A) In reverse direction the thermionic current of an ideal Schottky diode rises linearly when the applied voltage exceeds -0.1V due to the Schottky effect. No sublinear behavior is expected. (B) Tunneling current rises superlinearly in reverse direction.

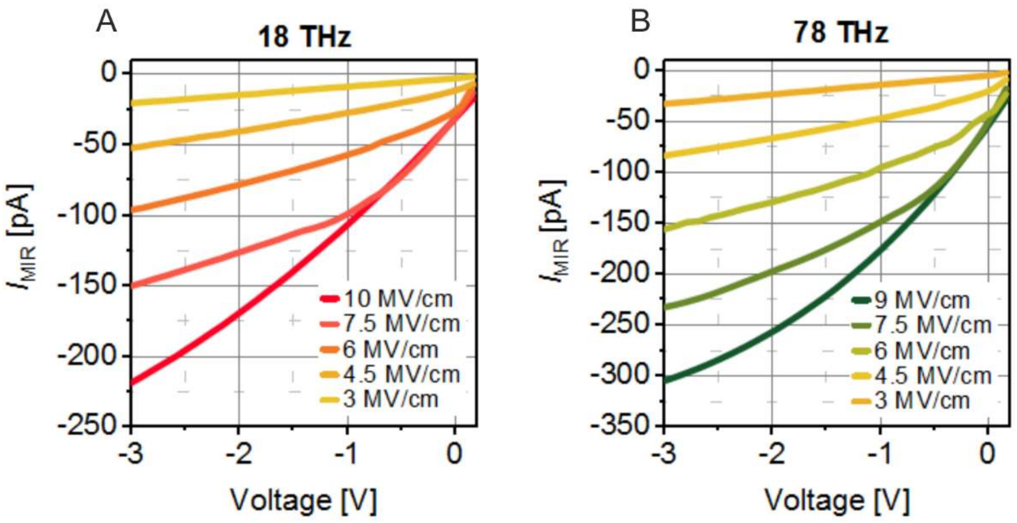

Fig. S5: Modelled MIR induced current at 18 THz and at 78 THz: The curves show again a sublinear like voltage dependency matching the experimental data (cf. Fig. 4).

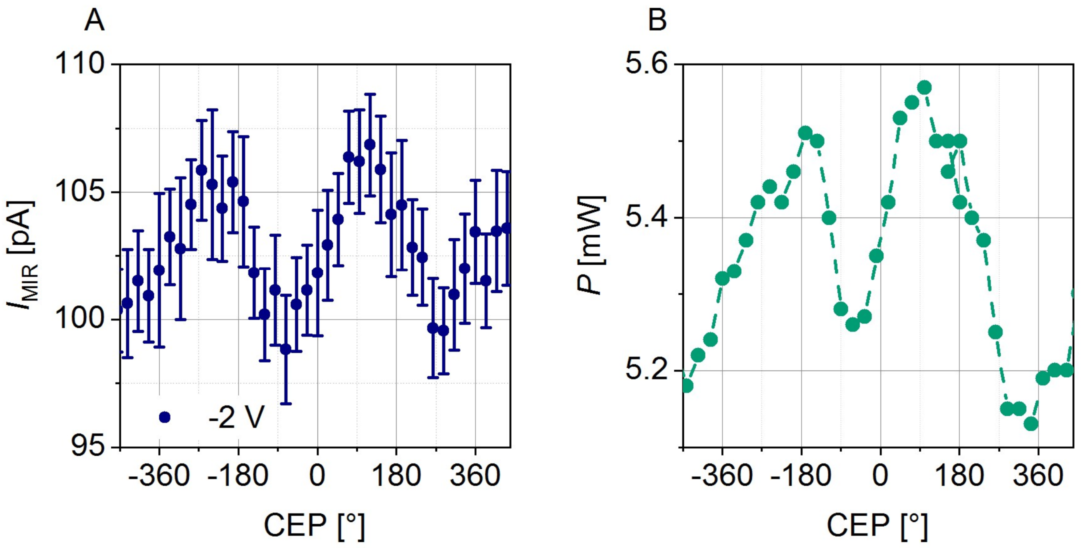

Fig. S6: Measured CEP dependency of I_{MIR}: (A) The MIR induced current at 18 THz showed a clear CEP dependency. (B) Dependency of the mean power of the MIR pulses as a function of the CEP.

**parameter**	**value**
A^*	17 ± 3 A K^{-2} cm^{-2}
ϕ	0.35 ± 0.02 eV
n	1.25 ± 0.01
N	$1.6 \cdot 10^{17} \pm 0.8 \cdot 10^{17}$ cm^{-3}

Table S1: Parameters of the Schottky diode extracted from the DC IV characteristics shown in Fig. S1.
